# Evidence of Autoimmune-Related Effects of Trichloroethylene Exposure from Studies in Mice and Humans

**DOI:** 10.1289/ehp.11782

**Published:** 2009-01-09

**Authors:** Glinda S. Cooper, Susan L. Makris, Paul J. Nietert, Jennifer Jinot

**Affiliations:** 1 National Center for Environmental Assessment, U.S. Environmental Protection Agency, Washington, DC, USA;; 2 Medical University of South Carolina, Charleston, South Carolina, USA

**Keywords:** autoimmune liver disease, solvents, systemic sclerosis, trichloroethylene

## Abstract

**Objective:**

Our objective was to examine experimental and epidemiologic studies pertaining to immune-related, and specifically autoimmune-related, effects of trichloroethylene (TCE).

**Data sources and extraction:**

We performed a literature search of PubMed and reviewed bibliographies in identified articles. We then systematically reviewed immune-related data, focusing on clinical and immunologic features and mechanistic studies.

**Data synthesis:**

Studies conducted in MRL^+/+^ lupus mice report an accelerated autoimmune response in relation to exposure to TCE or some metabolites. Effects have been reported after 4 weeks of exposure to TCE at doses as low as 0.1 mg/kg/day in drinking water and have included increased antinuclear antibodies and interferon-γ (IFN-γ) and decreased secretion of interleukin-4 (IL-4), consistent with an inflammatory response. Autoimmune hepatitis, inflammatory skin lesions, and alopecia have been found after exposures of 32–48 weeks. Recent mechanistic experiments in mice examined oxidative stress and, specifically, effects on lipid-peroxidation–derived aldehydes in TCE-induced autoimmune disease. Two studies in humans reported an increase in IL-2 or IFN-γ and a decrease in IL-4 in relation to occupational or environmental TCE exposure. Occupational exposure to TCE has also been associated with a severe, generalized hypersensitivity skin disorder accompanied by systemic effects, including hepatitis. In three case–control studies of scleroderma with a measure of occupational TCE exposure, the combined odds ratio was 2.5 [95% confidence interval (CI), 1.1–5.4] in men and 1.2 (95% CI, 0.58–2.6) in women.

**Conclusion:**

The consistency among the studies and the concordance between the studies in mice and humans support an etiologic role of TCE in autoimmune disease. Multisite collaborations and studies of preclinical immune markers are needed to further develop this field of research.

Trichloroethylene (TCE) is an industrial solvent that has been used extensively in industrial operations involving metal cleaning and degreasing. Its metabolism through a cytochrome P450 (CYP) pathway involving the enzyme CYP2E1 results in numerous metabolites, including chloral, chloral hydrate, dichloroacetic acid, trichloracetic acid, trichloroethanol, and trichloroethanol glucuronide (Lash et al. 2000). Many studies of immune-related effects of TCE have been conducted in the past decade, with much of this work focusing on autoimmune disease. We reviewed this recent research to determine the strength and consistency of data from experimental and epidemiologic studies, and the concordance between human and animal data, pertaining to these effects.

## Materials and Methods

We searched PubMed (National Center for Biotechnology Information 2008) for studies related to TCE and immune effects, using forms of “immune,” “autoimmune,” “immunosuppression,” “cytokines,” “lupus,” “scleroderma,” “rheumatoid arthritis,” “vasculitis,” and “liver” as search terms. We also reviewed references within relevant reports, including the National Academy of Sciences 2006 report on TCE (National Research Council 2006). We did not include studies of *in utero* or developmental exposures (Blossom and Doss 2007; Blossom et al. 2007; Peden-Adams et al. 2006, 2008) in this review.

We abstracted data pertaining to immunosuppression, hypersensitivity, and autoimmune-related effects, including clinically expressed or clinically diagnosed disease, cytokine expression or levels, T-cell activation, and serology [antinuclear antibodies (ANA) and specific autoantibodies]. We present, in tabular form, end points that all, or almost all, of the identified reports examined and include information on both “positive” and “negative” effects found in the studies.

We identified three case–control studies of scleroderma (systemic sclerosis) risk that included a specific assessment of occupational exposure to TCE in addition to more general assessments of “solvent” or “organic solvent” exposure (Diot et al. 2002; Garabrant et al. 2003; Nietert et al. 1998). We created graphical displays of the estimated measures of association from these studies using Comprehensive Meta Analysis, version 2.2.046 (Biostat, Inc., Englewood, NJ). We conducted a meta-analysis of these studies using a random-effects model to include the possibility of nonrandom error between studies (DerSimonian and Laird 1986). Studies were too sparse to do sensitivity analyses or explore sources of heterogeneity or potential publication bias.

## Results

### Immunosuppression Studies

Several animal studies have examined immunosuppression and host resistance in relation to TCE exposures by various routes and concentrations of exposure. CD1 mice exhibited an increase in susceptibility to infectious agents (e.g., *Streptococcus zooepidimicus*, *Klebsiella pneumoniae*) with short-term (3-hr) inhalation TCE exposures ranging from 2.6 to 48 ppm (Aranyi et al. 1986). Another study in female CD1 mice provided evidence of decreased humoral immunity with oral exposure (via drinking water) to TCE at concentrations of 0.1, 1, 2.5, or 5 mg/mL for 3–6 months (Sanders et al. 1982). Kauffmann et al. (1982) found similar results in a study of male and female CD1 mice exposed to a TCE metabolite, chloral hydrate, at concentrations of 0.07 and 0.7 mg/mL. After 3 days of single intra peritoneal (ip) injections of TCE in Sprague-Dawley rats at 0.05, 0.5, or 5 mmol/kg/day and B6C3F_1_ mice at 10 mmol/kg/day, natural killer cell activity in the liver, but not the spleen, was depressed in rats given the highest dose (22% lower than control levels, *p* < 0.05); in mice, a smaller decrease was reported (14% lower than control levels), which was not statistically significant (Wright et al. 1991).

Data pertaining to measures of immunosuppression (e.g., infectious disease risk) in humans are very limited. In 1979, testing of wells in Woburn, Massachusetts, revealed that the water in two of the wells was contaminated with a number of solvents, including TCE (267 ppb) (Lagakos et al. 1986). In 1982, Lagakos et al. (1986) used a telephone survey of Woburn residents (representing ~ 57% of the town residences with listed telephone numbers) to collect information on residential history and history of 14 types of medically diagnosed conditions for 4,978 children born since 1960 who lived in Woburn before they reached 19 years of age. Using exposure information based on estimates of the contribution of water from the contaminated wells in various zones within the town, Lagakos et al. (1986) estimated a cumulative exposure based on each child’s length of residence in Woburn. They found that a higher cumulative exposure measure was associated with a history of kidney and urinary tract disorders (primarily kidney or urinary tract infections) and with lung and respiratory disorders (asthma, chronic bronchitis, or pneumonia).

### Studies of Generalized Hypersensitivity Skin Diseases with Other Systemic Effects

Occupational exposure to TCE has been associated with a severe, generalized skin disorder that is distinct from contact dermatitis in its clinical presentation (which often involves mucosal lesions) and in the accompanying systemic effects, which can include lymphadenopathy, hepatitis, and other organ involvement. In two studies investigating delayed hypersensitivity using the guinea pig maximization test, sensitization rates of 66–71% and skin edema and erythema were observed (Tang et al. 2002, 2008). Elevations in liver enzymes and presence of liver lesions were also reported by Tang et al. (2008). In the guinea pig maximization test they found histopathologic evidence of fatty degeneration in the liver, hepatic sinusoid dilation, and inflammatory cell infiltration with an acute intradermal dose of 4,500 mg/kg, and diffuse ballooning without lymphocytic infiltration or necrotic hepatocytes with a total dosage of ≤ 340 mg/kg.

Kamijima et al. (2007) reviewed case reports describing 260 patients, mostly from Asia, with TCE-related generalized skin disorders. Their review is based on 26 articles published between 1966 and 2005 describing cases seen in China, Korea, Japan, Philippines, Singapore, Thailand, Spain, and the United States. One study in Guandong province, in southeastern China, included > 100 cases in a single year (Huang et al. 2002). Kamijima et al. (2007) categorized case descriptions as indicative of hypersensitivity syndrome (*n* = 124) or a variation of erythema multiforme, Stevens-Johnson syndrome, and toxic epiderma necrolysis (*n* = 115), with 21 other cases unclassified in either category. Hepatitis was common (> 50%), and the fatality rate (~ 10%) was similar in the two groups. Based on the reports reviewed by Kamijima et al. (2007), generalized skin disease within a work-site occurred in 0.25–13% of workers in the same location performing the same type of work. The measured concentration of TCE ranged from < 50 mg/m^3^ to > 4,000 mg/m^3^, and exposure scenarios included inhalation only and inhalation with dermal exposures. Disease manifestation generally occurred within 2–5 weeks of initial exposure, with some intervals of up to 3 months. Most of the reports were published after 1995, and the geographic distribution of cases reflects the newly industrializing areas within Asia. An analysis of breathing zone concentrations of volatile organochlorines and metal contaminants of the solvents used in factories of 25 workers hospitalized for hyper sensitivity skin disease and at similar factories with no affected workers in the past 3 years indicated no commonality of additives or impurities detected among the affected factories that could explain the occurrence of the hypersensitivity disorder (Kamijima et al. 2008).

### Autoimmune Disease Studies

#### Autoimmune mouse models

##### Acceleration of autoimmune response

Several mouse strains spontaneously develop conditions resembling systemic lupus erythematosus seen in humans. These strains have been extensively used in mechanistic research pertaining to disease pathogenesis. The MRL-*lpr* and NZB × NZW mouse strains exhibit extremely high disease penetrance and early death. The MRL^+/+^ strain is less severely affected, with disease development after 12 months (compared with 6–8 months in MRL-*lpr* and female NZB × NZW mice) and a relatively low prevalence of renal involvement. The MRL^+/+^ mouse has been used most often in experimental studies of TCE exposure (Table 1).

Several studies in MRL^+/+^ mice reported autoimmune-related effects from exposure to TCE via drinking water (Blossom et al. 2004, 2007; Cai et al. 2008; Gilbert et al. 1999; Griffin et al. 2000a, 2000b, 2000c; Wang et al. 2007b) or ip injection (Cai et al. 2006; Khan et al. 1995) (Table 1). The initial drinking water study used relatively high TCE concentrations of 2.5 and 5 mg/mL, with serologic measurements of ANA and IgG levels and assays of activation of CD4^+^ T cells from splenocytes (Gilbert et al. 1999; Griffin et al. 2000a). Another study examined lower exposure levels (0.1, 0.5, and 2.5 mg/mL) and extended the observation to 32 weeks (Griffin et al. 2000b). These studies demonstrated an acceleration of the autoimmune response. T-cell activation peaked at 4–8 weeks in the higher-dose experiment and at 32 weeks in the lower-dose experiment. The higher dose study showed evidence of a reversal in effects at 22 weeks, with lower levels of CD4^+^ T-cell expression and interferon-γ (IFN-γ) in exposed mice compared with controls. The effects observed by Griffin et al. (2000a) with respect to formation of TCE–protein adducts and CD4^+^ T-cell activation were blocked in a parallel study of 2.5 mg/mL TCE via drinking water in which the CYP2E1 metabolic pathway was inhibited by the addition of dial-lyl sulfide (Griffin et al. 2000c). In another longer-exposure duration study using one dose group (0.5 mg/mL TCE in drinking water), Cai et al. (2008) found no evidence of systemic inflammation as determined by serum cytokines measured after 36–48 weeks of exposure: levels of two proinflammatory cytokines, interleukin-6 (IL-6) and tumor necrosis factor-α (TNF-α), were decreased, but there was no difference in IL-1α.

Several studies demonstrated the involvement of one or more potential metabolites of TCE in the autoimmune response seen in MRL^+/+^ mice. These include studies of dichloroacetyl chloride (Khan et al. 1995, 2001), trichloroacetaldehyde hydrate (Blossom et al. 2004, 2007), and trichloroacetic acid (Blossom et al. 2004) (Table 1). Effects were similar to those found with TCE in terms of accelerated autoantibody expression, T-cell activation, and secretion of inflammatory cytokines, but some differences in clinical expression were found in the long-term studies. B-cell activation has not been observed in studies using TCE (Griffin et al. 2000a) or its metabolites (Blossom et al. 2004).

##### Chronic effects

The three chronic oral exposure studies in the MRL^+/+^ mouse, with exposure periods of 32–48 weeks, reported the presence of distinct clinical effects in exposed mice (Table 1). One of these effects was characterized as an auto immune hepatitis (Cai et al. 2008; Griffin et al. 2000b). Griffin et al. (2000b) found an inflammatory hepatic infiltrate in the portal tracts and lobular focal areas in the 0.5- and 2.5-mg/mL groups, with a dose-related effect on severity scores seen at 32 weeks. Alanine aminotransferase was also increased in the 0.5 mg/mL TCE dose group (means, 38.2 and 20.0 IU/L for 0.5 mg/mL and control groups, respectively). Cai et al. (2008) found similar liver lymphocytic infiltrates at 36 weeks and 48 weeks in a study using 0.5 mg/mL TCE exposure in drinking water, and infiltrates in the pancreas, lungs, and kidneys at 48 weeks. In a 40-week study using trichloroacetaldehyde hydrate, Blossom et al. (2007) found clinical effects, including diffuse alopecia and skin inflammation and ulceration, with increasing incidence across the dose groups; the difference between the 0.9 mg/mL group and the controls was statistically significant. Histologic examination of the skin lesions showed mono nuclear cell infiltration, mast cell hyperplasia, and dermal fibrosis.

A chronic (26-week) drinking water exposure study in NZB × NZW mice reported an increased level of proteinuria and prevalence of renal pathology, but no evidence of liver disease, with a TCE exposure of 10,000 ppb in drinking water (Gilkeson et al. 2004) (Table 1). Production of anti–double-stranded DNA (anti-dsDNA) and other antibodies was increased at 1,400 ppb TCE (but not 14,000 ppb) at 19 weeks; the differences lessened from 23 to 30 weeks and then increased between 32 and 34 weeks.

In a study in the male MRL-*lpr/lpr* mouse, Kaneko et al. (2000) exposed the mice via inhalation for 8 weeks to 0, 500, 1,000, or 2,000 ppm TCE (4 hr/day, 6 days/week). IgG and IgM levels were reduced, but IgA levels and levels of T-helper and T-suppressor cells did not differ across exposures over the course of the study. The authors also found histologic changes in the liver and spleen, but not in the thymus, in the highest dose group (Table 1).

##### *In vitro* and mechanistic studies

Initial studies of TCE exposure in MRL^+/+^ mice noted the activation of a specific immune serum antibody response directed against dichloroacetylated proteins (Gilbert et al. 1999; Griffin et al. 2000a; Khan et al. 1995). Immunohistochemical staining also revealed TCE-modified protein adducts in the liver and lung (Gilbert et al. 1999; Griffin et al. 2000a). Cai et al. (2007) extended these observations using an experiment involving immunization of various albumin adducts (trichloroethene oxide, dichloroacyl, formyl, and trifluroacyl) injected subcutaneously for 0, 2, or 4 weeks in groups of five female MRL^+/+^ mice. The authors used trifluroacyl albumin adduct as a positive control based on its role in halothane autoimmune liver disease. The immunogenicity of the protein adducts was demonstrated by the increased albumin-specific IgG seen in all albumin-adduct–treated groups. Cross-reactivity between adducts was also evident. Although Cai et al. (2007) found no differences in serum liver enzymes (alanine aminotransferase and aspartate aminotransferase), the formyl-albumin immunization resulted in a hepatic lymphocyte infiltration. These results suggest that the production of formyl-haptenized proteins from TCE may be a key factor in the development of the effects seen in the MRL^+/+^ mouse studies.

A series of experiments examined the role for oxidative stress, and specifically of lipid-peroxidation-derived aldehydes, in TCE-induced autoimmune disease. The hypothesized mechanism involves the binding of these reactive aldehydes to proteins to form neoantigen targets for autoantibody formation. Wang et al. (2007a) detected malondialdehyde and 4-hydroxynonenal protein adducts (reflecting lipid peroxidation) in the livers of female MRL^+/+^ mice treated for 6 and 12 weeks with 10 mmol/kg TCE (every fourth day, by ip injection). The same group found antimalondialdehyde anti bodies and antihydroxynonenal antibodies with 4- to 12-week ip exposure to TCE (Khan et al. 2001; Wang et al. 2007a, 2007b, 2008) and 48-week oral exposure to TCE (Wang et al. 2007b) or dichloroacetyl chloride (Khan et al. 2001). These antibodies were correlated with ANA (Wang et al. 2007a, 2007b). Wang et al. (2008) found proliferation and activation of splenic CD4^+^ T cells after stimulation with malondialdehyde-adducted or 4-hydroxy-nonenal–adducted mouse serum albumin.

Other studies have examined potential mechanisms through which TCE-related compounds affect T-cell stimulation and apoptosis. In a short-term (4-week) exposure study, Blossom et al. (2004) found a dose-related inhibition of anti-CD3–mediated (activation-induced) apoptosis of CD4^+^ T cells with oral exposures to 0, 0.1, and 0.9 mg/mL trichloroacetaldehyde hydrate. With a longer exposure period (40 weeks), however, they found no effect on anti-CD3–mediated apoptosis of CD4^+^ T cells and no difference in expression of Fas or Fas ligand (Blossom et al. 2007). This difference paralleled a difference in metalloproteinase-7 levels, which were elevated at 4 weeks but not at 40 weeks (Blossom et al. 2007; Blossom and Gilbert 2006).

#### Other rodent models

The Brown Norway rat has been used as a model of chemical-induced complex-mediated glomerulonephritis, for example, in studies of mercuric chloride (Sapin et al. 1977). The disease is complex-mediated, similar to that seen in humans with systemic lupus erythematosus. Polyclonal stimulation and increased IgE levels are hallmarks of the disease in the Brown Norway rat. White et al. (2000) administered TCE by gavage to female Brown Norway rats for 6 weeks (100, 200, or 400 mg/kg, 6–8 animals per dose) and found no increase in IgE compared with the vehicle controls.

In the study of NZB × NZW mice described above, Gilkeson et al. (2004) also included a parallel study of B6C3F_1_ mice, a model that typically does not develop auto-immune sequelae. Increases in anti-dsDNA antibodies were statistically significant or of borderline significance (*p* = 0.07) at all times after 26 weeks exposure to 1,400 ppb or 14,000 ppb TCE via drinking water.

### Epidemiologic Studies

#### Measures of immune function and markers

The available data from studies in humans also provide evidence of an association between increased TCE exposure and cytokine measures indicative of an inflammatory immune response (Table 2). Iavicoli et al. (2005) examined cytokine levels in 35 male TCE-exposed workers (degreasers) from a printing area of a factory in Italy and compared these workers with two control groups: other male factory workers not involved in degreasing activities (*n* = 30) and male office workers at the factory (*n* = 40). The mean TCE concentration among exposed workers was approximately 35 mg/m^3^. The authors observed no difference in serum cytokine concentrations (measured using enzyme-linked immunosorbent assays) between the two control groups, but they found considerable differences in the exposed group compared with the nonexposed workers (for all, *p* < 0.01): exposed workers had lower IL-4 levels and higher IL-2 and IFN-γ levels (Table 2).

Two other studies measured the percentage of cytokine-secreting CD3^+^ (or CD3^+^ and CD8^+^) T cells in relation to measures of TCE exposure from indoor air samples. Lehmann et al. (2001) found no association in the pilot study conducted with 28 children 36 months of age, but a larger study of 85 infants (4 weeks of age) reported a decrease in the population of IL-4–secreting cells and an increase in IFN-γ–secreting cells in relation to higher exposure to TCE (defined as > 75th percentile of the distribution) (Lehmann et al. 2002).

Byers et al. (1988) collected serum samples from 23 family members of leukemia patients in Woburn. They used these samples to assess the presence of autoantibodies (ANA, anti-smooth muscle, anti ovarian, anti thyroglobulin, and antimicrosomal antibodies) in the family member samples and compared the results with laboratory reference values. None of the serum samples from family member contained antithyroglobulin or antimicrosomal antibodies, but ANA were detected in samples from 10 (43%) family members (compared with < 5% expected based on the reference value).

The aquifer of the Santa Cruz River in Tucson, Arizona, had been contaminated with solvents (primarily TCE) and heavy metals (e.g., chromium). Kilburn and Warshaw (1992, 1993) examined the prevalence of connective tissue disease symptoms and ANA, comparing 362 residents of Tucson to 158 residents of another area of southwest Arizona. They selected the Tucson residents from the neighborhoods with documented water contamination (> 5 ppb TCE for at least 1 year between 1957 and 1981). The recruitment process was not described clearly, but in Tucson it seems to have specifically involved patients with lupus or other rheumatic diseases (Kilburn and Warshaw 1992, 1993). In contrast, they recruited the comparison group through a Catholic parish. The prevalence of some self-reported symptoms (malar rash, arthritis/arthalgias, Raynaud syndrome, skin lesions, and seizure or convulsion) and of low-titer (1:80) ANA was higher in Tucson (Kilburn and Warshaw 1992). It is difficult to attribute these differences to the water supply, however, given the process that went into participant recruitment (with part of the Tucson group selected based on the presence of rheumatic diseases) and the lack of verification of the self-reported symptoms.

#### Systemic autoimmune diseases

Interest in the role of chlorinated compounds in auto-immune diseases was spurred by the observation of a scleroderma-like disease in workers exposed to vinyl chloride (Gama and Meira 1978), and several case reports focused on TCE (Flindt-Hansen and Isager 1987; Lockey et al. 1987; Saihan et al. 1978). Larger epidemiologic studies (case–control or population-registry–based designs) using broad measures of occupational exposure to solvents, organic solvents, or chlorinated solvents generally reported a 2- to 3-fold increased risk of systemic sclerosis (scleroderma) (Aryal et al. 2001; Garabrant et al. 2003; Maitre et al. 2004), rheumatoid arthritis (Lundberg et al. 1994; Sverdrup et al. 2005), undifferentiated connective tissue disease (Lacey et al. 1999), and anti-neutrophil-cytoplasmic antibody (ANCA)–related vasculitis (Beaudreuil et al. 2005; Lane et al. 2003), but similar associations have not been found in studies of lupus (Cooper et al. 2004; Finckh et al. 2006).

Two case–control studies of scleroderma (Bovenzi et al. 2004; Maitre et al. 2004) and two of rheumatoid arthritis (Olsson et al. 2000, 2004) provided data concerning solvent exposure that occurred among metal workers or in jobs that involved cleaning metal (i.e., types of jobs that were likely to use TCE as a solvent). Olsson et al. (2000, 2004) found a 2-fold increased risk among male workers in studies of rheumatoid arthritis from Sweden. The results from the smaller studies of scleroderma were more variable: Maitre et al. (2004) found no exposed cases in a study with 93 cases and 206 controls, and Bovenzi et al. (2004) found an odds ratio (OR) of 5.2 [95% confidence interval (CI), 0.7–37] in a study with 56 cases and 171 controls.

**Figure 1:**
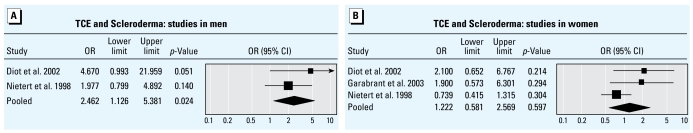
Association between risk of scleroderma and occupational exposure to TCE in men (*A*) and women (*B*), based on data from three case–control studies (Diot et al. 2002; Garabrant et al. 2003; Nietert et al. 1998). The graphics show the estimated measures of association from these studies. The pooled estimates are based on a meta-analysis using a random-effects model to include the possibility of nonrandom error between studies. Each square and corresponding line represent the OR and 95% CI from the individual study, with the relative size of the squares reflecting the relative weights of the studies. The horizontal midpoint of each diamond represents the pooled OR estimate and the horizontal extremes indicate the 95% CI.

Five other case–control studies provided data specifically about TCE exposure, based on industrial hygienist review of job history data (Table 3). Three of these studies are of scleroderma (Diot et al. 2002; Garabrant et al. 2003; Nietert et al. 1998), one is of undifferentiated connective tissue disease (Lacey et al. 1999), and one is of small vessel vasculitis involving ANCA (Beaudreuil et al. 2005). These studies included some kind of expert review of job histories, but only two studies included a characterization of exposure level (e.g., a cumulative exposure metric) or a “high” exposure group (Diot et al. 2002; Nietert et al. 1998). Most of the studies presented data stratified by sex. The test for heterogeneity between results for males and those for females just missed statistical significance (*p* = 0.058). We decided that performing separate analyses for these groups was warranted because the substantial gender-related differences in the background rate of scleroderma and in the prevalence of TCE exposure provide additional rationale for examining gender-related differences in measures of association. In men, the studies generally reported ORs between 2.0 and 8.0, and in women, the ORs were between 1.0 and 2.0. The meta-analysis of scleroderma studies with TCE exposure data (Diot et al. 2002; Garabrant et al. 2003; Nietert et al. 1998) resulted in a combined OR for “any” exposure of 2.5 (95% CI, 1.1–5.4) for men and 1.2 (95% CI, 0.58–2.6) for women (Figure 1). There was no clear support for heterogeneity within the male and female subgroupings (*p* = 0.35 for men and 0.16 for women).

## Discussion

During the past decade there has been considerable research, in experimental animals and in humans, pertaining to immune-related effects of TCE. Data concerning immunosuppression, particularly in humans, are still somewhat limited. In contrast, a fuller picture is shown by the studies of hypersensitivity, dermatitis, and hepatic injury, an emerging occupational concern in China and other industrializing areas.

The autoimmune-related studies, many of which were conducted in the past 5 years, represent a broad and robust literature, exhibiting many of the attributes that lend support for a causal relation between exposure and disease, including strength, consistency, dose–response patterns, and biological plausibility (Hill 1965; Legator and Morris 2003). Autoimmune mouse strains with a relatively less severe lupus phenotype (i.e., delayed disease onset), such as seen in the MRL^+/+^ mouse, provide an experimental model to examine the acceleration of lupus pathology. Numerous studies using different routes of exposure and dose ranges have demonstrated an accelerated autoimmune response in MRL^+/+^ mice. In the oral exposure studies, the duration of exposure period clearly influences the observed effects, with the acceleration (as measured by auto-antibody production, T-cell activation, and inflammatory cytokine secretion or serum levels) generally seen with shorter exposures (Blossom et al. 2004; Griffin et al. 2000a, 2000b), and clinical effects seen with longer exposure periods (Blossom et al. 2007; Cai et al. 2008; Gilkeson et al. 2004; Griffin et al. 2000b). The different effects seen with short compared with long exposure durations in the MRL^+/+^ mouse experiments may reflect the varying disease course and varying response in specific target tissues during different phases of pathogenesis (Greidinger et al. 2007). It is interesting that the clinical effects seen in the MRL^+/+^ mouse chronic exposure studies (36–48 weeks) (Blossom et al. 2007; Cai et al. 2008; Griffin et al. 2000b) differ somewhat from “normal” expression within these mice. That is, the effects seen included an inflammatory hepatic infiltrate, diffuse alopecia, and skin inflammation and ulceration.

Studies using autoimmune mouse strains also provide a means to examine potential mechanisms of action as well as the effects of various doses and durations of exposure. The formation of immunogenic TCE-induced adducts of liver proteins and lipid-peroxidation–derived aldehydes and potential role of oxidative stress in promoting an aberrant autoimmune response to neoantigens in the liver and other tissues are some of the mechanistic hypotheses that have been raised by this research.

Data from experimental studies in non-lupus-prone animals are limited. In a study in the Brown Norway rat, White et al. (2000) found no increase in IgE with gavage doses of up to 400 mg/kg-day for 6 weeks, but Gilkeson et al. (2004) found increases in anti-dsDNA antibodies in B6C3F_1_ mice after 26 weeks of exposure to 1,400 ppb or 14,000 ppb via drinking water.

Are the effects seen in the MRL^+/+^ mouse also seen in humans? Occupational studies have examined inflammatory cytokines and specific systemic autoimmune diseases, although our literature search did not find any studies of TCE exposure and either lupus or autoimmune liver disease. We found no cohort studies of the incidence of auto immune diseases in workers exposed to TCE, but the available case–control studies of scleroderma and TCE support the relevance of this disease within the context of human exposures. Although the association is generally stronger in studies in men compared with those in women, it is not clear whether this difference reflects the relatively low background risk of scleroderma in men, differences in exposure prevalence or in the reliability of exposure assessment (Messing et al. 2003), or a gender-related difference in susceptibility to the effects of TCE. Given the available studies in scleroderma and more limited data in other systemic autoimmune diseases, it is difficult to argue that the investigations into disease acceleration and variations in manifestations of effect seen in lupus-prone mice are irrelevant to humans. The variation in clinical expression of disease in response to TCE exposure remains an important research question to be addressed by both mechanistic and epidemiologic studies.

Systemic sclerosis (scleroderma) and autoimmune liver disease are relatively rare in humans, with incidence rates of about 1/100,000 per year; incidence rates for systemic lupus erythematosus are somewhat higher (5–10 per 100,000 per year) (Cooper and Stroehla 2003) but still low enough to present considerable challenges to performing adequately powered epidemiologic studies. An additional impediment to autoimmune disease research is the lack of disease registries, which adds to the difficulty in identifying incident cases of specific diseases. The insights generated by the recent experimental studies of TCE, however, provide a strong rationale for developing the type of multi site collaborations needed to address the potential influence of TCE or other solvents operating through a similar pathway on the incidence of specific autoimmune diseases, and for evaluating the potential immune-related effects of low-level exposures from environmental contamination. Development and validation of exposure assessment techniques incorporating occupational and environmental exposure pathways, use of quantitative exposure metrics, examination of multiple solvents and other agents, and studies of markers of immune function expressed before onset of disease would be instrumental in the further advancement of these efforts.

## Figures and Tables

**Table 1:** Studies of TCE and metabolite effects^a^ in lupus mouse strains by strain, exposure route, and duration.

Strain, reference(s), exposure	Serology	Studies of cultured splenocytes	Clinical/histopathology results
Female MRL^+/+^ mice, drinking water			

Gilbert et al. 1999; Griffin et al. 2000a No. per group; 0, 2.5, 5 mg/mL TCE (average 0, 455, 734 mg/kg-day) for 4, 8, or 22 weeks	Increased ANA at 4 and 8 weeks; no difference between groups at 22 weeks	Increased activated CD4^+^ T cells and IFN-γ secretion across doses at 4 weeks, with effects reversed at 22 weeks; decreased IL-4 secretion (4 and 22 weeks)	No evidence of liver or renal damage, based on serum alanine aminotransferase, sorbitol dehydrogenase, and blood urea nitrogen
Griffin et al. 2000c 8 per group; 0, 0.1, 0.5, or 2.5 mg/mL TCE (0, 21, 100, or 400 mg/kg-day) for4 or 32 weeks	Increased ANA in all treated groups at 4 weeks but not at 32 weeks	Increased activated CD4^+^ T cells (32 weeks) and IFN-γ secretion (4 and 32 weeks); no effect on IL-4 secretion	Extensive hepatic mononuclear cellular infiltrate in 0.5 and 2.5 mg/mL groups; hepatocyte reactive changes in all treated groups at 32 weeks
Blossom et al. 2004 6–8 per group; 0, 0.1, 0.9 mg/mL trichloroacetaldehyde hydrate (0, 24, 220 mg/kg) or trichloroacetic acid (0, 27, 205 mg/kg-day) for 4 weeks	Increased ANA and anti-histone antibodies at 0.9 mg/mL trichloroacetaldehyde hydrate^b^	Increased activated CD4^+^ T cells at 0.1 and 0.9 g/mL doses of both metabolites; increased IFN-γ secretion at 0.9 mg/mL; no effect on IL-4 secretion	No evidence of liver or kidney damage, based on serum alanine aminotransferase and liver and kidney histology
Blossom et al. 2007 8 per group, 0, 0.1, 0.3, 0.9 mg/mL trichloroacetaldehyde hydrate (0, 13, 46, 143 mg/kg-day) for 40 weeks	Slightly suppressed anti-ssDNA, anti-dsDNA, and anti-histone antibody expression; differences not statistically significant	Increased activated CD4^+^ T-cells and increased IFN-γ secretion; no effect on IL-4 secretion	Diffuse alopecia, skin inflammation and ulceration, mononuclear cell infiltration, mast cell hyperplasia, and dermal fibrosis; statistically significant increase at 0.9 mg/mL dose group but also increased at lower doses; no liver or kidney histopathology effects seen
Cai et al. 2008 5 per group, 0 or 0.5 mg/mL TCE (mean 60 μg/g-day) for 48 weeks	Increased ANA after 24 weeks but not statistically significant	Increased IFN-γ secretion after 36 weeks but not statistically significant	Hepatic necrosis; hepatocyte proliferation; leukocyte infiltrate in the liver, lungs, and kidneys; no difference in serum aminotransferase liver enzymes

Female MRL^+/+^ mice, ip injection			

Khan et al. 1995 4–5 per group, in corn oil, 0 (corn oil), 10 mmol/kg TCE, 0.2 mmol/kg dichloroacetyl chloride, every 4th day for 6 weeks	In both groups, increased ANA and anti-ssDNA antibodies; in dichloroacetyl chloride group, anticardiolipin antibodies; no difference in anti-histone, -Sm, or -DNA antibodies	Not evaluated	Not evaluated
Cai et al. 2006 6 per group, in corn oil, 0 (corn oil), 0.2 mmol/kg dichloroacetyl chloride, 0.2 mmol/kg dichloroacetic anhydride, twice per week for 6 weeks	In both treated groups, increased ANA	In both treated groups, increased IL-1α, IL-1β, IL-3, IL-6, IFN-γ, granulocyte colony–stimulating factor, and keratinocyte-derived chemokine secretion and decreased IL-5; in dichloroacetyl chloride group, increased IL-17 and IFN-α^c^	In both treated groups, increased lymphocytes in spleen and thickening of alveolar septa with lymphocytic interstitial infiltration

Female NZB × NZW mice, drinking water			

Gilkeson et al. 2004 6 per group, 0, 1,400, or 14,000 ppb,^d^ 26 weeks exposure; observed up to 36 weeks	Increased anti-dsDNA antibodies at some periods at ≥ 1,000 ppb	Not evaluated	At 10,000 ppb, proteinuria increased beginning at 20 weeks; renal pathology scores increased; no signs of liver disease

Male MRL –*lpr/lpr*, inhalation			

Kaneko et al. 2000 5 per group, 0, 500, 1,000, 2,000 ppm TCE, 4 hr/day, 6 days/week for 8 weeks	Not evaluated	Not evaluated	At ≥ 500 ppm, dose-related liver inflammation, splenomegaly, and hyperplasia of lymphatic follicles; at 1,000 ppm, immunoblastic cell formation in lymphatic follicles; no changes in thymus

ssDNA, single-strand DNA.

a Selected end points based on those reported across most studies.

b No difference reported in anti-dsDNA, anti-ss-DNA, anti-ribonucleosome, anti-SSA, anti-SSB, anti-Sm, anti-Jo-1, or anti-Scl-70 antibodies.

c No difference in secretion of other cytokines measured: IL-2, IL-4, IL-10, IL-12, TNF-α, granulocyte monocyte colony–stimulating factor, macrophage inflammatory protein-1α, and RANTES (CCL-5).

d Dose not given in mg/kg-day. Dose levels cited by Gilkeson et al. (2004) were incorrectly reported as 1,000 and 10,000 ppb; corrected doses (Peden-Adams MM, personal communication) are reported here.

**Table 2:** Studies of cytokines and TCE exposure in humans.

Reference, location, no., and age	Source of exposure data, outcome measures	Results
Iavicoli et al. 2005 Italy; 35 exposed workers, 30 nonexposed workers, and 40 office worker controls, all male; mean age ~ 33 years	Urine sample (for trichloroacetic acid concentration); workplace TCE measures (personal samples, 4 exposed and 4 nonexposed workers); questionnaire (smoking history, age, residence); serum cytokine levels	Nonexposed workers similar to office controls for all cytokine measures; compared with nonexposed workers, TCE-exposed workers had decreased IL-4 (mean, 3.9 vs. 8.1 pg/mL), increased IL-2 (mean, 798 vs. 706 pg/mL), and increased IFN-γ (mean, 37.1 vs. 22.9 pg/mL)
Lehmann et al. 2001 Germany; 28 children, 36 months of age	Indoor air sampling (child’s bedroom) of 28 volatile organic chemicals; cytokine-secreting CD3^+^ and CD8^+^ T cell populations from blood sample	TCE exposure not associated with percentages of IL-4 CD3^+^ or IFN-γ CD8^+^ T-cells
Lehmann et al. 2002 Germany; 85 newborns	Indoor air sampling (child’s bedroom) of 28 volatile organic chemicals (4 weeks after birth); cytokine-secreting CD3^+^ T cell populations from CD3^+^ cord blood cells	Increasing TCE levels associated with decreased IL-4 (OR = 4.4; 95% CI, 1.1–17.8) for < 25th percentile IL-4 and increased IFN-γ (OR = 3.6; 95% CI, 0.9–14.9) for > 75th percentile IFN-γ; no association with TNF-α or IL-2

**Table 3:** Epidemiologic studies of TCE exposure and risk of scleroderma and other autoimmune diseases.

Disease, reference, location, period, sample size, age	Source of exposure data	Exposure definition/prevalence			
		Source of exposure data	Cohort	Cases (%)	Controls (%)	OR (95% CI)
Scleroderma (systemic sclerosis)					

Nietert et al. 1998 South Carolina; prevalent cases, 1995–1997; 178 cases (141 women, 37 men), 200 hospital-based controls; mean age at onset, 45.2 years	Structured interview (specific jobs and materials; jobs held ≥ 1 year); classified by expert review (job exposure matrix)	Men			
		Any exposure^a^	51	42	2.0 (0.8–4.9)
		Maximum intensity	30	10	3.3 (1.0–10.3)
		Cumulative intensity	32	21	2.0 (0.7–5.3)
		Maximum probability	16	3	5.1 (NC)
		Women			
		Any exposure^a^	19	24	0.7 (0.4–1.3)
		Maximum intensity	6	7	0.9 (0.3–2.3)
		Cumulative intensity	10	9	1.2 (0.5–2.6)
		Maximum probability	4	5	0.7 (0.2–2.2)
Diot et al. 2002 France; prevalent cases hospitalized 1998–2000; 80 cases (69 women, 11 men), 160 hospital controls; mean age at diagnosis, 48 years	Structured interview (specific jobs and materials; jobs held ≥ 6 months); classified by expert review	Men and women			
		Any exposure	16	8	2.4 (1.0–5.2)
		High exposure^b^	9	1	7.6 (1.5–37.4)
		Men			
		Any exposure	64	27	4.7 (0.99–21.9)
		Women			
		Any exposure	9	4	2.1 (0.65–6.8)
Garabrant et al. 2003 Michigan and Ohio; prevalent cases diagnosed 1980–1992; 660 cases (all women), 2,227 population controls;^c^ ages, ≥ 18 years	Structured interview (specific jobs and materials; jobs held ≥ 3 months); classified by self-report and expert review	Women			
		Self-report, any exposure	1.3	0.7	2.0 (0.8–4.8)
		Expert review, any exposure	0.7	0.4	1.9 (0.6–6.6)

Undifferentiated connective tissue disease					

Lacey et al. 1999 Michigan and Ohio; prevalent cases diagnosed 1980–1991 (Michigan), 1980–1992 (Ohio); 205 cases (all women), 2,095 population controls; ages, ≥ 18 years	Structured interview (specific jobs and materials; jobs held ≥ 3 months); classified by self-report and by expert review	Women			
		Self-report, any exposure	0.5	0.7	0.88 (0.11–6.95)
		Expert review, any exposure	0.5	0.4	1.67 (0.19–14.9)

ANCA-related diseases^d^					

Beaudreuil et al. 2005 France; incident cases diagnosed 1999–2000; 60 cases (~ 50% women), 120 hospital controls; mean age, 61 years	Structured interview (specific jobs and materials; jobs held ≥ 6 months); classified by expert review	Men and women			
		Any exposure	18.3	17.5	1.1 (0.5 – 2.4)
		Data not presented separately by sex			

NC, not calculated.

a The publication did not include “any” exposure, but this was generated by the original author (P. Nietert) as part of the present analysis.

b Product of probability × intensity × frequency × duration scores, summed across all jobs; scores of > 1 classified as “high.”

c Total number with TCE data: self-report 606 cases, 2,138 control; expert review 606 cases, 2,137 controls.

d Diseases included Wegener glomerulonephritis (*n* = 20), microscopic polyangiitis (*n* = 8), pauci-immune glomerulonephritis (*n* = 10), uveitis (*n* = 6), Churg-Strauss syndrome (*n* = 4), stroke (*n* = 4), and other diseases (≤ 2 each).
